# Heterogeneous neural coding of corrective movements in motor cortex

**DOI:** 10.3389/fncir.2013.00051

**Published:** 2013-04-04

**Authors:** Adam S. Dickey, Yali Amit, Nicholas G. Hatsopoulos

**Affiliations:** ^1^Committee on Computational Neuroscience, University of ChicagoChicago, IL, USA; ^2^Department of Statistics, University of ChicagoChicago, IL, USA; ^3^Department of Organismal Biology and Anatomy, University of ChicagoChicago, IL, USA

**Keywords:** double-step, reaching, motor cortex, neural coding, target jump

## Abstract

During a reach, neural activity recorded from motor cortex is typically thought to linearly encode the observed movement. However, it has also been reported that during a double-step reaching paradigm, neural coding of the original movement is replaced by that of the corrective movement. Here, we use neural data recorded from multi-electrode arrays implanted in the motor and premotor cortices of rhesus macaques to directly compare these two hypotheses. We show that while a majority of neurons display linear encoding of movement during a double-step, a minority display a dramatic drop in firing rate that is predicted by the replacement hypothesis. Neural activity in the subpopulation showing replacement is more likely to lag the observed movement, and may therefore be involved in the monitoring of the sensory consequences of a motor command.

## INTRODUCTION

There is a long tradition of investigating motor control by using a double-step reaching paradigm, where a target jumps to a new location after a movement is initiated (Georgopoulos et al., 1981; Soechting and Lacquaniti, 1983; Goodale et al., 1986; Pelisson et al., 1986; Paulignan et al., 1991; Prablanc and Martin, 1992). For example, this double-step paradigm was used to implicate posterior parietal cortex (PPC) in monitoring the error between the hand and target position, because online corrections are not made in response to a double-step when this area is inactivated by transcranial magnetic stimulation (Desmurget et al., 1999; Reichenbach et al., 2011) or by a lesion (Grea et al., 2002).

Only a few studies have used extracellular recordings in cerebral cortex to investigate neural coding during a double-step reaching paradigm (Georgopoulos et al., 1983; Archambault et al., 2009, 2011). These studies found that in primary motor (MI) and dorsal premotor (PMd) cortices (Archambault et al., 2011), and in area 5 of the PPC (Archambault et al., 2009), neural activity during a double-step was well-explained by replacing the original neural activity with neural activity corresponding to the correction elicited by the target jump.

However, this idea of replacement is at odds with the traditional view in the motor cortical encoding literature, which describes neural activity during reaching as a linear function of hand kinematics, particularly the instantaneous direction and speed (Georgopoulos et al., 1982; Schwartz et al., 1988; Moran and Schwartz, 1999; Wang et al., 2007). These models would not predict anything different during a double-step reach.

Here, we use multi-electrode arrays to record neural data from MI, PMd, and ventral premotor (PMv) cortices from rhesus macaques performing reaches, and directly compare the default, linear encoding hypothesis to the replacement hypothesis previously proposed in cortical double-step studies. The replacement hypothesis (Archambault et al., 2009, 2011) was developed in the context of a standard center-out task, requiring reaches from a center target to one of eight peripheral targets arranged in a circle. The double-step involved jumping from the original target to a target located 180^°^ opposite on the circle. Double-step activity was fit by starting with the original neural activity for movement from the center to the first target and replacing it with neural activity for movement from the center to the second target, which predicted the observed neural activity better than replacing it with neural activity associated with movement from the center to a randomly selected target (Archambault et al., 2009, 2011). Though these studies found that a linear encoding model generalized poorly from single-step to double-step trials (Archambault et al., 2009, 2011), the prediction of the “Replacement” hypothesis was not directly compared to the alternative of linear encoding of observed kinematics. This is because the “Replacement” prediction was derived from the directly observed, trial-averaged neural activity, instead of the prediction of a linear model fit using single-trials. Thus it is not clear that the replacement model predicts double-step neural activity better than a standard linear encoding model of observed kinematics.

Here, we use the concept of superposition to allow a direct comparison of the two hypotheses. It has been previously shown that the kinematics during a double-step can be expressed as the superposition (or vector sum) of the original, unperturbed movement and a corrective movement from the original target location to the new target location (Flash and Henis, 1991). We define the “Replaced” hypothesis to mean that, during a double-step trial, neurons first encode the kinematics of the original movement, and then switch to encode the kinematics of the corrective movement. To test this prediction, the observed movement first needs to be decomposed into a linear combination of its two constituent parts: the original movement and the corrective movement. To allow a direct and fair comparison to the standard linear encoding model, we define an alternative “Summed” hypothesis which states that neurons encode the summed kinematics of the two constituent movements, which should closely match the observed movement.

**FIGURE 1:**
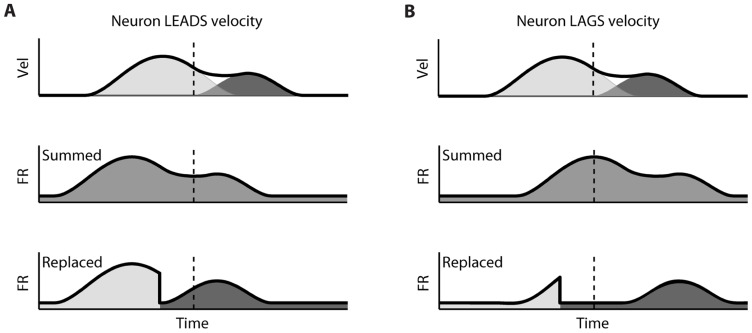
**Schematic of the two hypotheses for neural coding during a double-step**. **(A)** *Top*: The theoretical double-peaked velocity profile of a double-step trial (black) can be decomposed into the sum of a primary velocity profile (light gray) and an overlapping, secondary velocity profile (dark gray); *middle*: Under the default “Summed” hypothesis, neural firing is predicted to track the observed velocity profile, preceding it at a fixed lead; *bottom*: The alternative “Replaced” hypothesis predicts that the neural firing will track the primary velocity profile (light gray), before replacing this with coding of the secondary profile (dark gray). **(B)** The predictions under the “Summed” and “Replaced” hypotheses if neuronal firing instead lags the observed velocity. The replacement between coding of the primary and secondary movements is assumed to happen at a fixed delay that we call the *neural offset*, prior to the start of the second movement (dashed line).

The difference between the “Replaced” and “Summed” hypotheses can be best understood in the context of a double-step movement in one dimension, where the target is simply perturbed farther in the direction of the original movement (**Figure 1**). If the target jump happens soon after movement onset, the correction will be triggered before the original movement ends. This means that the velocity profile will be double-peaked, and it will not return to 0 between the peaks (**Figure 1A**, top). This double-peaked profile can be decomposed into the sum of two overlapping single-peaked speed profiles. If we assume that neurons linearly encode the velocity in the neuron’s preferred direction at a single leading time delay, then the firing rate profile should also be double-peaked (**Figure 1A**, middle). That is the “Summed” prediction. In contrast, in the “Replaced” prediction, the firing rate first follows the original, single-peaked profile and then switches to the second, corrective single-peaked profile at some time prior to the start of the second movement (**Figure 1A**, bottom). This switch produces a sharp drop in firing rate back to the baseline level, before rising again to match the corrective velocity profile.

If instead neuronal firing lags the velocity profile, the “Summed” prediction simply shifts to the right but is otherwise unchanged (**Figure 1B**, middle). However, if the switch time happens at the same time point, then the “Replaced” hypothesis predicts an extended silent period where the firing rate drops to the baseline level, before rising again to track the corrective movement (**Figure 1B**, bottom). The predictions of the “Summed” and “Replaced” hypotheses are quite different, so we should be able to resolve on a neuron by neuron basis which hypothesis better fits single-trial neural activity.

## MATERIALS AND METHODS

### NEURAL RECORDINGS

**FIGURE 2:**
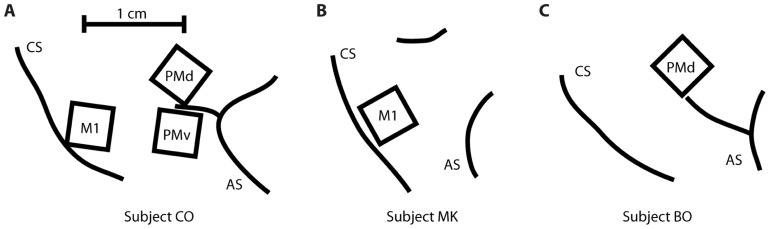
**Location of the five multi-electrode arrays relative to the central sulcus (CS) and the arcuate sulcus (AS)**. **(A)** Subject CO had arrays in primary motor cortex (MI), dorsal premotor cortex (PMd), and ventral premotor cortex (PMv). **(B)** Subject MK had one array in MI. **(C)** Subject BO had one array in PMd.

Three rhesus macaques (*Macaca mulatta*) were implanted with a total of five Utah 100-microelectrode arrays (Blackrock Microsystems, Salt Lake City, UT, USA) in MI, PMv, or PMd cortices in the right hemisphere (contralateral to the arm used for the task). Subject CO had arrays in MI, PMd, and PMv, subject MK had an array in MI, and subject BO had an array in PMd (**Figure 2**). The length of the electrodes on subject CO’s MI array was 1.5 mm, while the length on the other four arrays was 1 mm. All electrode tips were sputter-coated with platinum, except for subject MK’s MI electrode tips, which were coated with iridium oxide. The procedure for implanting the Utah array has been described elsewhere (Rousche and Normann, 1992; Maynard et al., 1999). During a recording session, signals from 96 electrodes were amplified (gain of 5,000), band-pass filtered between 0.3 Hz and 7.5 kHz, and recorded digitally (14-bit) at 30 kHz per channel using a Cerberus acquisition system (Blackrock Microsystems Inc., Salt Lake City, UT, USA). Only waveforms (duration, 1.6 ms; 48 sample time points per waveform) that crossed a voltage threshold were stored for off-line sorting. This voltage threshold was set just outside the noise band, so that all potential spike waveforms were recorded for later off-line spike sorting. Spike waveform data were sorted in Offline Sorter (Plexon, Dallas, TX, USA) using a user-defined unit template, which was a single waveform shape to which all potential spike waveforms were compared. All waveforms whose mean square error from this template fell below a user-defined threshold were classified as spikes belonging to that unit.

### BEHAVIORAL TASK

Subjects were operantly conditioned to perform a behavioral task requiring planar reaching movements using a two-link robotic exoskeleton (KINARM, BKIN Technologies, Kingston, ON, Canada) that sampled X and Y positions of the hand at 500 Hz. However, for these experiments, the shoulder angle was locked, so only one-dimensional, elbow flexion and extension movements were possible. Control (single-step) trials of the behavioral task involved maneuvering a cursor controlled by the hand position to acquire a target within 1,500 ms of its appearance and then holding on it for a random hold period (uniformly distributed from 300 to 700 ms). There was no instructed delay, so subjects were free to move to the target as soon as it appeared. There were five discrete target locations (numbered 1–5), equally spaced at 1.35, 1.5, 1.65, 1.8, and 1.95 radians, respectively. Here, 0 radians indicates a fully extended elbow and an increasing angle indicates elbow flexion. The target width was 0.05 radians. Since the subjects forearm lengths were approximately 20 cm, this corresponds to an approximately 1 cm wide target.

In addition to these single-step control trials, up to one-third of trials requiring movement between targets 2, 3, or 4 were perturbed to become double-step trials. The target shifted its position when the cursor moved more than 0.075 radians from the original target center.

In the three “Forward Jump” datasets, the target was perturbed in the same direction as the initial movement by 0.15 radians (for example, a movement starting at location 2 to a target at location 4 was perturbed by moving the target to location 5). In the four “Reverse Jump” datasets, the target was perturbed in the opposite direction of the initial movement by 0.15 radians. More details of the seven datasets analyzed are given below (**Table 1**). Of note, subject CO had all three arrays implanted simultaneously, and datasets #1 and #5 were recorded simultaneously. Each dataset represents all neurons recorded from one cortical area in a given day’s training session. All of the surgical and behavioral procedures were approved by the University of Chicago Institutional Animal Care and Use Committee and conform to the principles outlined in the *Guide for the Care and Use of Laboratory Animals.*

**Table 1:** Details are provided for the seven datasets reported here.

#	Area	Subject	Jump Dir.	Single	Double	Neurons	Analyzed
1	MI	CO	Forward	1,626	167	26	17
2	MI	MK	Reverse	1,276	228	70	41
3	MI	CO	Reverse	1,428	242	36	17
4	PMd	BO	Reverse	1,645	280	115	67
5	PMd	CO	Forward	1,626	167	88	30
6	PMd	CO	Reverse	1,407	299	128	56
7	PMv	CO	Forward	1,943	275	90	54

*From left to right, the columns list the dataset number (#), the cortical area recorded from (Area), the subject ID (Subject), whether the dataset had a “Forward” or “Reverse” double-step (Jump Dir.), the number of single-step trials (Single), double-step trials (Double), sorted neurons (Neurons), and neurons analyzed in the Section “Results” (Analyzed).*

### DATA PROCESSING

The raw angular position traces were first low-pass filtered forward and backward using a fourth order Butterworth filter and a 10-Hz cutoff frequency. These were then differentiated to obtain the angular velocity traces. For single-step trials, spike times were aligned on the first crossing of a 0.3-radians/s velocity threshold after target appearance. For double-step trials, spike times were aligned on the jump time (when the change in target location occurred). Mean trajectories and peri-event time histograms (PETHs) were computed by averaging kinematics and spike counts across all trials with the same starting and ending locations in a time window from -300 ms before the velocity threshold crossing (or jump time for the double-step trials) to 800 ms afterward.

### ENCODING MODEL

Previous studies have reported that motor cortical firing is linearly related to both the Cartesian velocity and speed of the hand at a single time lag (Moran and Schwartz, 1999), but also that cortical discharge is better explained by joint angular velocity than Cartesian velocity (Reina et al., 2001). For our 1-D behavioral task, we combine these two results and assume that during single-step trials motor cortical firing rate FR(*t*) is a linear function of the elbow joint angular velocity *V*(*t*) and speed | *V*(*t*)| at a single time delay δ. The baseline firing rate is *b*_0_, and *b*_1_ and *b*_2_ are the coefficients for velocity and speed tuning, respectively.

$$\begin{array}{cc} \text{FR(}t-\delta \text{)}=b_{0}+b_{1}V(t)+b_{2}|V(t)|. & \left(1\right) \end{array}$$

Although in 1-D angular velocity and speed can differ only in sign, they are uncorrelated and the linear prediction is better when using both. Spike times for each neuron from all successful single-step trials were binned every 10 ms, and the resulting spike counts were smoothed by convolving them with a Gaussian kernel with a standard deviation of 30 ms, similar to the Archambault et al.’s (2009; 2011) studies. These smoothed spike counts were fit as a linear function of the elbow angular velocity and speed, sampled every 10 ms and delayed by the parameter δ. We tested all possible delays from -300 to +300 ms in 10 ms increments, and kept the delay with the highest correlation coefficient between the predicted and actual binned spike counts. We also fit a logistic version of Eq. 1, using standard generalized linear model techniques, relating kinematics to binary spike counts in 10 ms bins (the 0.2% bins containing more than one spike were treated as having one spike).

Of the 553 neuron samples which were originally recorded across the seven datasets, we excluded from analysis the 190 neurons which had an encoding delay of greater than +175 ms. This is due to the fact that these neurons tended to respond precisely to the visual appearance of the target, usually 100 ms following target appearance (Reimer and Hatsopoulos, 2010), rather than in anticipation of future velocity or response to past velocity. For the remaining neurons, we compared the prediction of the encoding model (Eq. 1) to the mean PETHs computed for each combination of starting and end point by computing a correlation coefficient between the observed and predicted PETHs. We rejected an additional 81 neurons whose correlation coefficient was less than 0.5. This left 282 neurons for further analysis. The number of neurons analyzed in each dataset is given in **Table 1**. These are neurons for which the linear encoding model (Eq. 1) provides an adequate prediction of the firing rate on control, single-step motions between pairs of targets.

### DECOMPOSING DOUBLE-STEP KINEMATICS

It has been previously shown that kinematics during a target jump can be decomposed into the sum of two minimum jerk movements (Flash and Henis, 1991; Henis and Flash, 1995). If the inter-stimulus interval between the original target presentation and the target jump is greater than 100 ms, then the original movement is directed from the start point to the original target location, and the secondary movement is directed from the original target to the new target location (Henis and Flash, 1995).

We first fit the control, single-step movements to a single minimum jerk trajectory. The minimum jerk velocity profile is mathematically described below (Eq. 2) for a movement starting at time *t*_0_ with duration *d* and with an amplitude *a*, which is the change in position from the beginning to the end of the movement (Hogan, 1984; Flash and Hogan, 1985). Note that velocity is defined to be 0 before the start point *t*_0_ or after the endpoint *t*_0_ + *d*.

$$\begin{array}{cc} V(t;t_{0},a,d)=\left\{\begin{array}{l} 30\frac{a}{d}\left({\text{τ}}^{4}-2{\text{τ}}^{3}+{\text{τ}}^{2}\right),\text{τ}=\frac{\left(t-t_{0}\right)}{d}\text{for }t_{0}\leq t\leq t_{0}+d\,\\ \text{0otherwise} \end{array}\right\}. & \left(2\right) \end{array}$$

The angular velocity during a double-step trial *V*_J__UMP_ (*t*) was fit as a sum of a primary (*V*_1_) and secondary (*V*_2_) minimum jerk velocity profile (Eq. 3). Thus the double-step velocity can be described with six parameters.

$$\begin{array}{cc} \begin{array}{cc} V_{\text{JUMP}}(t)\approx V_{\text{SUM}}(t;t_{1},a_{1},d_{1},t_{2},a_{2},d_{2}) & \end{array} & \\ =V_{1}(t;t_{1},a_{1},d_{1})+V_{2}(t;t_{2},a_{2},d_{2}). & \left(3\right) \end{array}$$

We found the optimal set of six parameters to fit a given double-step velocity profile *V*_JUMP_(*t*) by minimizing the cost function expressed below (Eq. 4).

$$\begin{array}{cc} \begin{array}{l} \begin{array}{c} C(t_{1},a_{1},d_{1},t_{2},a_{2},d_{2})=\\ \frac{\left(1-\alpha \right)}{{\left({\text{σ}}_{\text{ERR}}\right)}^{2}}\left[\underset{t}{\Sigma }{\left(V_{\text{JUMP}}(t)-V_{\text{SUM}}(t;t_{1},a_{1},d_{1},t_{2},a_{2},d_{2})\right)}^{2}\right]+ \end{array}\\ \text{α}\left[\overset{2}{\underset{i=1}{\Sigma }}{\left(a_{i}-a_{i}^{*}\right)}^{2}/{\left({\text{σ}}_{a}\right)}^{2}+\overset{2}{\underset{i=1}{\Sigma }}{\left(d_{i}-d_{i}^{*}\right)}^{2}/{\left(\sigma_{d}\right)}^{2}+{\left(t_{2}-t^{*}\right)}^{2}/{\left({\text{σ}}_{t}\right)}^{2}\right]. \end{array} & \left(4\right) \end{array}$$

We make the assumption that the parameters of two component movements will be similar to the corresponding single-step motions. Thus, for a double-step which starts at location 2 where the target jumps from location 4 to 5, we assume the primary motion *V*_1_ is similar to the single-step movement from 2 to 4, and the secondary motion *V*_2_ is similar to the single-step movement from 4 to 5. The values of the coefficients were constrained by including a squared error term relative to a reference value, multiplied by a scaling factor. These reference values and scaling factors were derived from single-step trials. We fit the velocity profiles from single-step trials to a minimum jerk velocity profile (Eq. 2) by minimizing the sum of squared errors. The reference amplitudes $\left(a_{1}^{*},a_{2}^{*}\right)$ and durations $\left(a_{1}^{*},a_{2}^{*}\right)$ were set to the median of the amplitudes and durations fit to the corresponding single-step trials, and so these values varied from dataset to dataset. The remaining parameters (t*,σ*_a_*, σ*_b_*, σ*_t_*, σ_ERR_) were set constant for all datasets, and were derived from the “well-fit” single-step trials from one dataset (#7) whose correlation between actual and fit velocity was above 0.9 for 1,694 of 1,943 trials (87%). The start time of the first movement was unconstrained, but the reference start time of the second movement (relative to the jump time) was set as the mean of the reaction time of these well-fit single-step trials (t* = 220 ms), with scaling factor given by their standard deviation (σ*_t_* = 50 ms). Similarly, the scale factor for the amplitude and duration was again set to the pooled estimate of their standard deviations for the well-fit single-step trials (σ*_a_* = 0.3 radians, σ*_d_* = 90 ms). The sum squared error between the actual and fit velocity was normalized by the mean squared error of the well-fit single-step trials (σ_ERR_ = 0.08 radians/s). There was also an arbitrary weight constant (α = 0.95) added to prevent the velocity error term from dominating the coefficient error terms.

### “SUMMED” vs. “REPLACED” HYPOTHESIS

Under the default “Summed” hypothesis, the same encoding model (Eq. 1) that was fit to single-step trials was applied to double-step trials. However, rather than applying this encoding model to the observed velocity profile *V*_JUMP_, the firing rate was predicted using the fit velocity profile *V_SUM_*:

$$\begin{array}{cc} {\text{FR}}_{\text{SUM}}(t-\text{δ})=b_{0}+b_{1}V_{\text{SUM}}(t)+b_{2}\left|V_{\text{SUM}}(t)\right|. & \left(5\right) \end{array}$$

The “Summed” prediction is based on the fit velocity *V*_*SUM*_ to allow a direct comparison to the alternative “Replaced” hypothesis, where neurons instead encode the constituent movements *V*_1_ and *V*_2_ which comprise *V*_*SUM*_. The “Replaced” hypothesis states that neurons will first encode the primary movement, and then switch to encoding the secondary movement at some time after the target jump (Eq. 6). We assume that this switch time is fixed to the start of the second movement *t*_2_ minus some constant neural offset *t*_N_. To prevent over-fitting, we fixed the neural offset to a constant for each cortical area before comparing the prediction to the “Summed” hypothesis.

$$\begin{array}{cc} {\text{FR}}_{\text{REPLACE}}(t)=\left\{\begin{array}{c} b_{0}+b_{1}V_{1}(t+\text{δ})+b_{2}\left|V_{1}(t+\text{δ})\right|\;\text{for}\;t<t_{2}-t_{N}\\ b_{0}+b_{1}V_{2}(t+\text{δ})+b_{2}\left|V_{2}(t+\text{δ})\right|\;\text{for}\;t\geq t_{2}-t_{N} \end{array}\;\right\}. & \left(6\right) \end{array}$$

We compared the “Summed” (Eq. 5) and the “Replaced” (Eq. 6) predictions in terms of their fit to observed neural activity during a double-step trial. We predicted the spike count in 10 ms bins for each trial, and assessed the goodness of fit of these predictions by computing the root-mean-square error (RMSE) between the prediction and the smoothed spike train (after convolution with a Gaussian kernel with a 30-ms standard deviation). The calculation of the RMSE metric was limited to a time interval from the jump time to 500 ms after the jump, as the predictions of the two hypotheses were very similar outside of this window. The RMSE metric includes data, from double-step trials from all the movement conditions. Predicted firing rates less than 1 spike/s were replaced with replaced with 1 spike/s. This thresholding was performed to prevent the linear model from predicting a negative or zero firing rate. A paired *t*-test was then used to determine if the mean RMSE was significantly different between the two predictions.

We also compared the predictions for the “Summed” and “Replaced” hypotheses using the logistic version of Eq. 1. For the logistic prediction of firing rate, FR*(*t*), we computed a log-likelihood of observed binary spike counts in 10 ms bins, given the “Summed” or “Replaced” predictions. We assume spike counts are conditionally independent Bernoulli random variables. If the binary spike count for a given neuron at time *t* is denoted *x*(*t*), then the log-likelihood of a spike train given the “Summed” hypothesis (*L*_*SUM*_) is given in Eq. 7. A similar expression holds for log-likelihood of the “Replaced” hypothesis (*L*_*REPLACED*_).

$$\begin{array}{cc} L_{\text{SUM}}=\underset{t}{\Sigma }x\left(t\right)\log\left(\text{F}{\text{R}}_{\text{SUM}}^{*}\left(t\right)\right)+\left(1-x\left(t\right)\right)\log(1-\text{F}{\text{R}}_{\text{SUM}}^{*}\left(t\right)).\; & \left(7\right) \end{array}$$

## RESULTS

**FIGURE 3:**
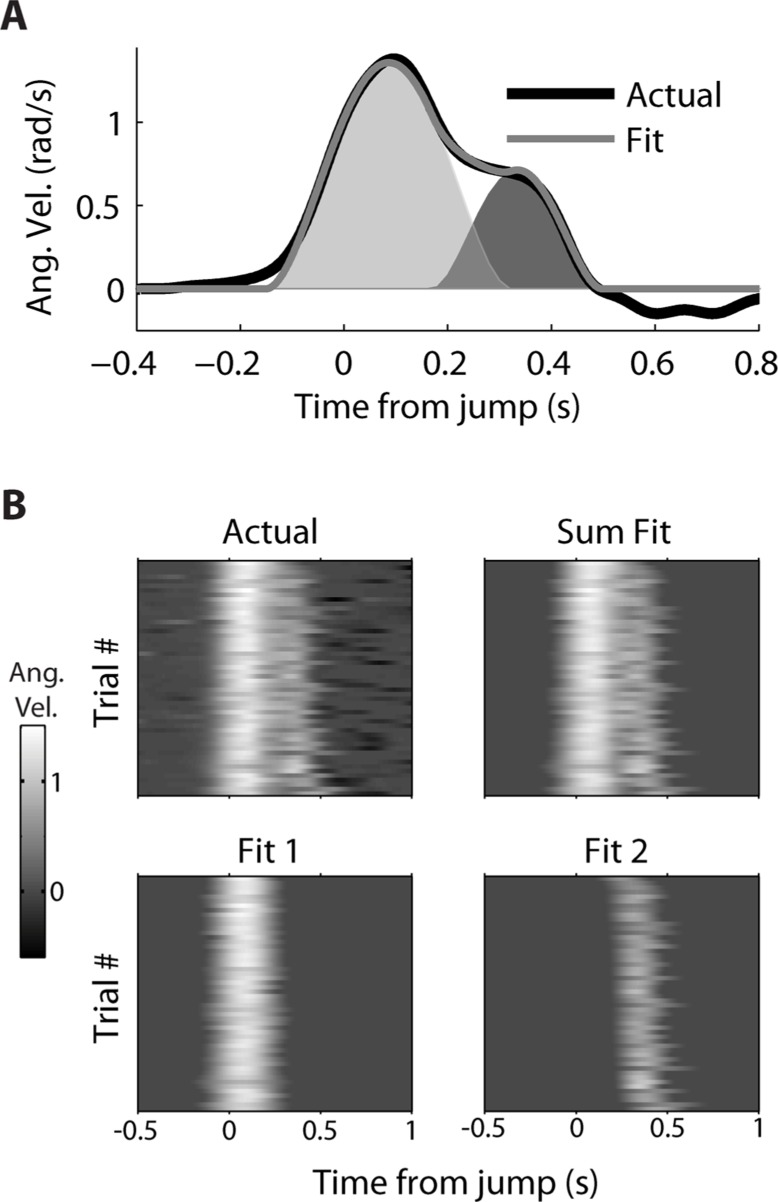
**Decomposition of actual kinematics observed during a double-step**. **(A)** The actual kinematics of a double-step trail (black line) are well-approximated by the sum (gray line) of a primary (light gray) and secondary (dark gray) single-peaked profiles. **(B)** The single-trial velocity profiles are displayed as heat maps, with the vertical axis representing different trials, and the horizontal axis representing time elapsed within a given trial. The actual kinematics (top left) can be compared to the fit kinematics (top right), which is the sum of the single-trial primary (bottom left) and secondary (bottom right) motions.

A decomposition algorithm was used (see Decomposing Double-step Kinematics) to fit the double-step velocity profiles to the sum of two minimum jerk velocity profiles (**Figure 3**). For a single trial (**Figure 3A**), the fit velocity profile does a good job matching the bulk of the observed double-peak profile, though it does not fit the reversal in velocity, which comes after the second movement. The fraction of variance (*R*^2^) of the actual velocity explained by the fit velocity was 0.986. The decomposition performed similarly well for all 52 double-step trials for this movement condition from dataset #7 (**Figure 3B**), where the starting point was location 2, and the target jumped from location 4 to 5. The main difference is that the fit velocity is constant at 0 before and after the double-peaked velocity profile. The median *R*^2^ for these trials was also 0.986, and the mean was 0.983 (standard deviation 0.015). For the 1,658 successful double-step trials from all datasets, the median *R*^2^ was 0.982, with a mean of 0.970 (standard deviation 0.0357).

We can use the parameters of the fit velocity profile as an estimate of the reaction time, either from the target appearance or the target jump (**Table 2**, left). The average reaction time of all single-step trials (with amplitude 0.15 radians) of a given dataset varied from 203 to 230 ms. However, when looking across the seven datasets, the mean of these single-step reaction times was not significantly different from the mean of the first or second double-step reaction times (paired *t*-test, *p* > 0.05). The average movement duration for single-step trials (with amplitude 0.15 radians) varied from 405 to 457 ms (**Table 2**, right). Similarly, when looking across the seven datasets, the mean of these single-step durations was not significantly different from the mean of the first or second double-step durations (paired *t*-test, *p* > 0.05).

**Table 2:** The mean and standard deviation of the reaction times (RT) and movement durations of the single-step (SS), the first double-step (DS 1), and the second double-step (DS 2) movements for all seven datasets.

#	RT mean (± SD) in ms			#	Duration mean (± SD) in ms		
	SS	DS 1	DS 2		SS	DS 1	DS 2
1	228 (47)	207 (40)	222 (34)	1	416 (66)	418 (68)	366 (51)
2	203 (71)	202 (57)	201 (50)	2	457 (102)	469 (83)	446 (82)
3	231 (53)	224 (42)	181 (48)	3	405 (71)	399 (61)	416 (85)
4	221 (84)	253 (46)	248 (44)	4	406 (105)	404 (100)	409 (119)
5	228 (47)	207 (40)	222 (34)	5	416 (67)	418 (68)	366 (51)
6	230 (55)	221 (40)	174 (43)	6	413 (71)	396 (51)	413 (77)
7	223 (50)	208 (43)	217 (39)	7	430 (76)	420 (55)	389 (84)

*Data are reported for all single-step trials with an amplitude of 0.15 radians, and all double-step trials where each component has an amplitude of 0.15 radians. Outlying reaction times >400 ms (6% of original data) and durations >700 ms (7%) were removed before the mean and standard deviation were computed.*

Given the decomposition, we still need one extra parameter to predict the neural firing under the “Replaced” hypothesis: the neural offset *t_N_*. This is a constant which represents how soon before the start of the secondary movement (*t*_2_) the neurons show the replacement effect. We first assumed that the neural offset for each neuron was constant for all movement conditions, but different across neurons. We then tested a range of offsets for each neuron, from 0 to 250 ms in 10 ms increments, and picked the offset which minimized the RMSE of the data given the “Replaced” prediction. Within this range, we are trying to find a change in neural activity in the reaction time period, between the change in target position but before the onset of movement. We then compared the distributions of neural offsets across cortical regions, from neurons which were better fit by the “Replaced” prediction (lower mean RMSE, *p* < 0.05, *t*-test) and whose neural offset was greater than 0 ms and less than 250 ms.

**FIGURE 4:**
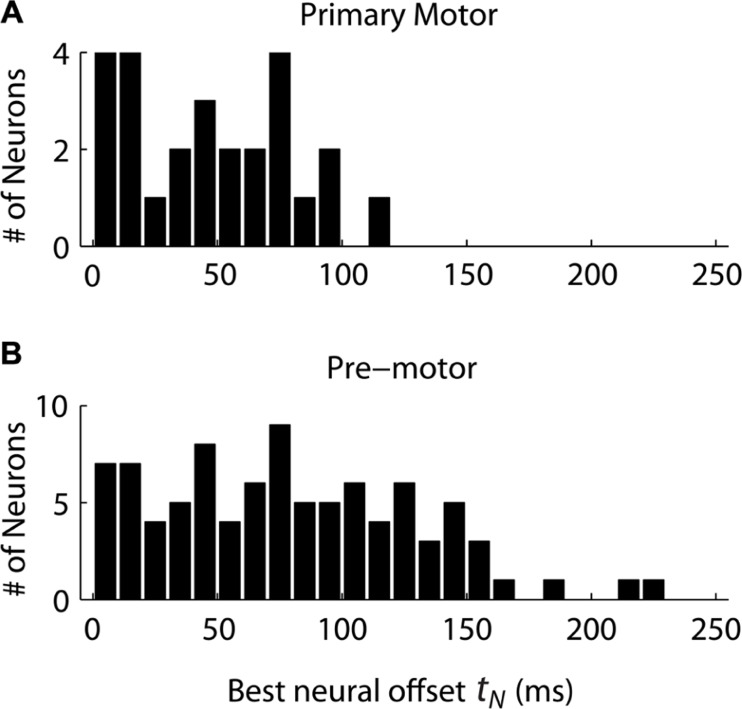
**The distribution of neural offsets is compared between neurons from the primary motor cortex **(A)** and the premotor cortex **(B)**, including both dorsal and ventral premotor cortices**. The neural offset describes the time at which the “Replaced” hypothesis predicts a shift from coding the primary movement to coding the secondary movement (see **Figure 1**).

The distribution of neural offsets was markedly different between MI and premotor cortices, i.e., PMd and PMv cortices (**Figure 4**). The mean neural offset of the 26 eligible MI cortical neurons (53 ms) was significantly less (*p* < 0.01, *t*-test) than the mean of neural offset of the 91 eligible premotor neurons (84 ms). This indicates that premotor cortex shows an earlier response to the double-step target jump than MI cortex, consistent with previous results (Archambault et al., 2011). There was no significant difference in mean neural offsets between neurons in PMd and PMv (*p* > 0.05, *t*-test). To compare the “Replaced” prediction to the “Summed” prediction, we did not want the neural offset to be a free parameter, because this could lead to over-fitting of the “Replaced” neural prediction and would represent an unfair comparison between the two hypotheses. Thus, we rounded the mean neural offsets up to the nearest 10 ms, so that all MI neurons had a neural offset of 60 ms, and all premotor neurons had a neural offset of 90 ms. Recall that the neural offset specifies when the neural replacement happens relative to start of movement. It is different that the encoding delay, which specifies whether neural firing lags or leads observed movement.

**FIGURE 5:**
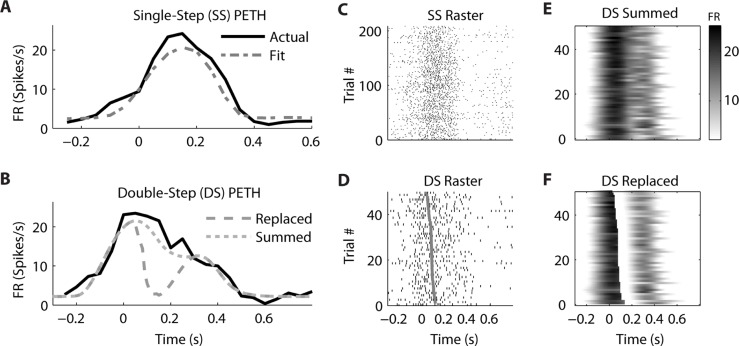
**A neuron from ventral premotor cortex is better fit by the “Summed” hypothesis**. **(A)** During single-step (SS) trials, the observed peri-event time histogram (PETH, black) is well fit by a model assuming linear encoding of velocity and speed (gray dot dash). Zero time refers to the time of target appearance. **(B)** During double-step (DS) trials, the observed PETH (black) is well fit by the “Summed” prediction (gray dotted) but not the “Replaced” prediction (gray dashed). Zero time refers to the time of the target jump. **(C)** The single-trial spike time rasters for the SS trials. **(D)** The single-trial spike time rasters for the DS trials, arranged by the predicted replacement time (gray). **(E)** The DS single-trial prediction of the “Summed” hypothesis, displayed as a heat map. **(F)** The DS single-trial prediction of the “Replaced” hypothesis.

We observed that the response profiles of some neurons were consistent with the “Summed” hypothesis while others were more consistent with the “Replaced” hypothesis during the double-step trials, even among neurons that were recorded simultaneously. For an example PMv neuron (from dataset #7), the prediction of a linear encoding model (fit on all single-step trials) closely matched the PETH for single-step movements from location 2 to 4 (**Figure 5A**). The *R*^2^ between the actual PETH (**Figure 5A**, solid black) and predicted PETH (**Figure 5A**, gray dot dash) was 0.96 for this movement condition. Fitting a linear encoding model to all single-step trials gave an optimal encoding delay δ of +30 ms, meaning this neuron’s firing led the observed velocity.

The corresponding double-step condition also involved starting at location 2 and moving to location 4, but the visual target was switched to location 5 after the original movement was initiated. This resulted in a double-peaked velocity (see **Figure 3**) where the constituent velocity profiles overlapped. For a neuron which leads velocity, the “Summed” hypothesis predicts the firing rate should follow the shape of the double-peaked profile, while the “Replaced” hypothesis predicts a temporary reset to baseline after the target jump (see **Figure 1A**). For this neuron, the “Summed” prediction much better fit to the observed double-step PETH than did the “Replaced” prediction (**Figure 5B**).

This neuron’s spike times from individual trials of the single-step (**Figure 5C**) and double-step (**Figure 5D**) conditions show a similar smooth rise and fall in firing rate. The raw double-step rasters (**Figure 5D**) can be visually compared to the single-trial predictions of the “Summed” (**Figure 5E**) and “Replaced” (**Figure 5F**) predictions. These are displayed as heat maps, where black indicates a high firing rate and white indicates a low firing rate. There is a sharp drop in firing rate (seen as a black to white transition) in the “Replaced” prediction (**Figure 5F**) around 100 ms after the target jump. However, this predicted drop in firing rate is not seen in the raw double-step rasters (**Figure 5D**) or the observed PETH (**Figure 5B**). Instead, the neuron reaches a similar peak firing rate as the single-step condition (23 spikes/s) at the jump time (0 s), before gradually decreasing its firing rate over the next 500 ms. Note that the double-step rasters and predictions (**Figures 5D–F**) are sorted by the start of the second constituent motion (as defined by the decomposition algorithm). This is why the reset to baseline is predicted to occur later for trials near the bottom for the “Replaced” hypothesis (**Figure 5F**). The *R*^2^ between the actual double-step PETH and the “Summed” prediction is 0.94, compared to 0.62 for the “Replaced” prediction. For this neuron, across all trials of all double-step conditions, the average RMSE for the “Summed” prediction (7.6 spikes/s) was significantly less (*p* < 0.001, paired-test) than the average RMSE of the “Replaced” prediction (8.2 spikes/s).

**FIGURE 6:**
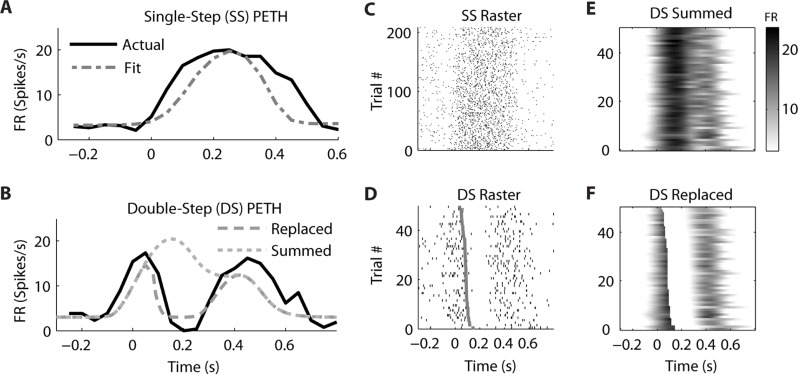
**A neuron from ventral premotor cortex is better fit by the “Replaced” hypothesis**. Format follows **Figure 5**.

In contrast, the firing of a different but simultaneously recorded PMv neuron was clearly better fit by the “Replaced” hypothesis (**Figure 6**). The single-step PETH (**Figure 6A**, black) from target 2 to 4 showed a smooth increase and decrease in firing rate. The firing rate profile was wider than that predicted by the linear encoding model (gray dot dash), but the *R*^2^ between the actual and fit PETH was still 0.80. This neuron’s optimal delay δ was -70 ms, indicating that neural firing lagged hand velocity. During the corresponding double-step profile, the “Summed” hypothesis failed to predict the neuron’s response (**Figure 6B**, gray dotted). It predicted that the neural firing rate should reach a peak of 20 spikes/s 200 ms after the target jump. Instead, the actual neural firing dropped below 2 spikes/s from 150 to 250 ms after the target jump. Unlike the “Summed” prediction, the “Replaced” prediction (gray dashed) successfully predicted this transient drop in firing rate for the double-step condition. The *R*^2^ between the actual and predicted double-step PETH was 0.80 for the “Replaced” hypothesis, but 0.05 for the “Summed” hypothesis.

While the single-trial spike times for the control, single-step trials (**Figure 6C**) showed a smooth increase and decrease in firing rate, each individual trial in the double-step condition showed an abrupt cessation of spiking activity (**Figure 6D**). This drop is not seen in the single-trial predictions of the “Summed” hypothesis (**Figure 6E**), but it is seen in the predictions of the “Replaced” hypothesis (**Figure 6F**). Across all trials of all double-step conditions, the mean RMSE for the “Replaced” prediction (7.5 spikes/s) was significantly less (*p* < 0.001, paired-test) than the mean RMSE of the “Summed” prediction (8.8 spikes/s).

**FIGURE 7:**
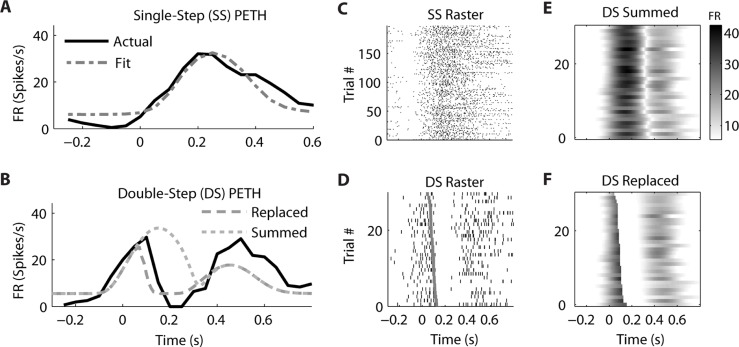
**A neuron from primary motor cortex is better fit by the “Replaced” hypothesis**. Format follows **Figure 5**.

**FIGURE 8:**
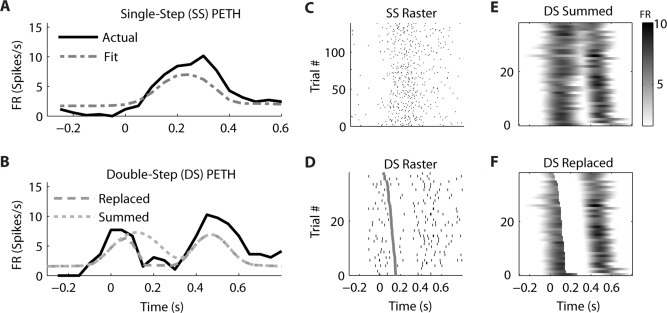
**A neuron from dorsal premotor cortex is better fit by the “Replaced” hypothesis**. Format follows **Figure 5**.

Similar examples of “Replaced” neurons can be found in MI (**Figure 7**) and PMd (**Figure 8**). The MI neuron (from dataset #2) had an optimal encoding delay δ of -90 ms, and the *R*^2^ between the actual and predicted single-step PETH for movement from location 2 to 4 was 0.86 (**Figure 7B**). The *R*^2^ between double-step PETH and the “Replaced” prediction was 0.68, and between the PETH and “Summed” prediction was 0.08. The RMSE was significantly less for the “Replaced” than the “Summed” prediction (14.2 vs. 15.7 spikes/s, *p* < 0.001, paired-test). In this dataset, the jump involved a reversal in the direction of movement (see Materials and Methods), but this neuron increased its firing rate for movements in both directions, so the “Replaced” prediction was similar to the previous example. The PMd neuron (from dataset #5) had an optimal encoding delay δ of -100 ms, and the *R*^2^ between the actual and predicted single-step PETH for movement from location 2 to 3 was 0.89 (**Figure 8B**). The *R*^2^ between double-step PETH and the “Replaced” prediction was 0.81, and between the PETH and “Summed” prediction was 0.40 (**Figure 8F**). The RMSE was significantly less for the “Replaced” than the “Summed” prediction (5.5 vs. 6.2 spikes/s, *p* < 0.001, paired-test).

Across all neurons, two-thirds were better fit with the “Summed” prediction, and one-third were better fit with the “Replaced” prediction (**Table 3**). For the linear version of Eq. 1, we defined the better prediction as having a lower average RMSE, while for the logistic version, the better prediction had a higher log-likelihood (see Materials and Methods). The percentage of cells which were better fit with the “Replaced” prediction (using RMSE) did not differ significantly between MI cortex (29/75, 39%) and premotor cortex (82/207, 40%).

**Table 3:** Neurons which were better “Replaced” were more likely to lag the kinematics (encoding delay <0) than neurons better “Summed.”

	Replaced	Summed	*p*-Value
**Linear**
Percentage of cells	39% (111)	61% (171)	
Percentage lagging	58% (64)	30% (66)	0.01
Mean delay	-49 ms	13 ms	<0.001
Median delay	-70 ms	30 ms	<0.001
**Logistic**
Percentage of cells	36% (108)	64% (194)	
Percentage lagging	56% (61)	43% (83)	0.02
Mean delay	-116 ms	-11 ms	0.01
Median delay	-140 ms	-10 ms	0.03

*The top rows list the percentage (and number) of cells better “Replaced” or better “Summed,” while the subsequent rows show the percentage of those cells with a lagging delay, the mean delay, and median delay. The percentages were compared using a chi-square test, the mean delays were compared with a *t*-test, and the median delays were compared with a Wilcoxon rank-sum test.*

**FIGURE 9:**
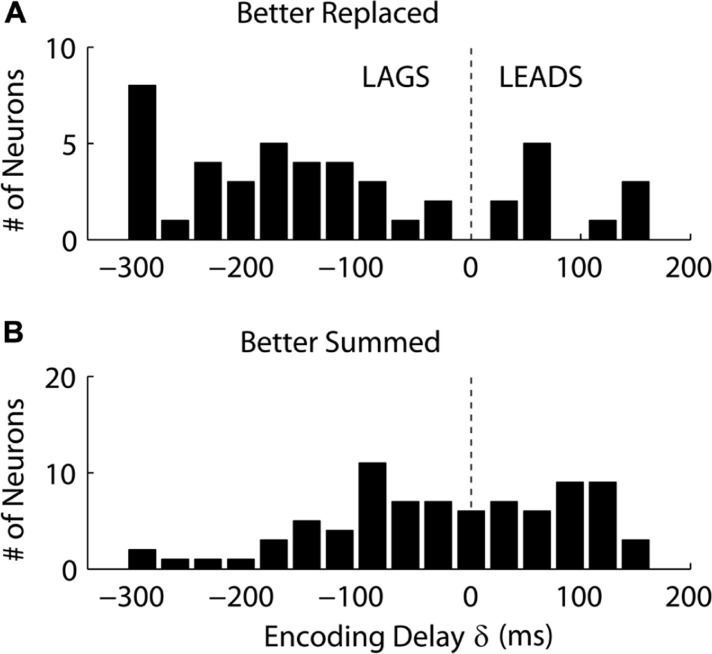
** The distribution of the encoding time delay δ is compared between neurons which were better fit with the “Replaced” hypothesis **(A)**, versus neurons better fit with the “Summed” hypothesis (B)**. Only neurons which showed a significant difference in single-trial root-mean-square error between the two hypotheses are shown (see Results for details).

Interestingly, we found that “Replaced” neurons were more likely to lag movement velocity while “Summed” neurons tended to lead movement velocity. Specifically, the percentage of neurons with an encoding delay δ less than 0 was significantly different for “Summed” and “Replaced” neurons, as were the mean and median lags (**Table 3**). Recall that the encoding lag is fit only to single-step trials, while the determination of “Replaced” vs. “Summed” is made on the double-step trials. This difference in the distribution of encoding delays was most dramatic when we looked at the 128 neurons that exhibited a significant difference (*p* < 0.01, *t*-test) in the mean RMSE between the “Summed” and “Replaced” predictions (**Figure 9**). Of these cells, 46 (36%) were better “Replaced” and 82 (64%) were better “Summed.” Again, the “Replaced” neuronal firing was significantly more likely (*p* < 0.001, chi-square test) to lag observed velocity (35/46 or 76% with δ < 0) than was “Summed” neuronal firing (42/82 or 51% with δ < 0).

## DISCUSSION

We found that for a third of the neurons analyzed from MI and premotor cortices, single-trial neural activity during a double-step was better explained with the “Replaced” rather than “Summed” encoding hypothesis. In some cases, the drop in firing rate predicted by the “Replaced” hypothesis was dramatic and readily visible in the single-trial raster plots (see **Figure 6**). Thus we were able to replicate the replacement phenomenon described previously (Georgopoulos et al., 1983; Archambault et al., 2009, 2011), though we saw it in only a minority of cells. The majority of neurons was consistent with the “Summed” hypothesis and conformed to the model of linear encoding of velocity (Schwartz et al., 1988; Moran and Schwartz, 1999).

Why did we only observe the “Replaced” phenomenon in a minority of cells? One potential difference from the studies by Archambault et al. (2009), 2011 is that we used a 1-D reaching paradigm rather than 3-D unconstrained reaching. We chose the 1-D paradigm to make the decomposition algorithm as simple as possible (see Decomposing Double-step Kinematics). However, we do not expect the change in the degrees of freedom to affect the results – the “Replaced” hypothesis was described for both 2-D planar reaching (Georgopoulos et al., 1983) and 3-D unconstrained reaching (Archambault et al., 2009, 2011). Likewise, linear encoding models have been used to describe motor cortical firing both for 2-D planar reaching (Moran and Schwartz, 1999) and 3-D unconstrained reaching (Wang et al., 2007).

We feel the difference from the Archambault et al.’s (2009; 2011) studies is simply one of methodology. The previous studies were documenting the existence of a phenomenon, so the “Replaced” hypothesis was compared to a null condition. This null condition replaced neural firing with a randomly selected firing pattern, while the alternative condition replaced with a firing pattern corresponding to the direction of the correction. That is an appropriate technique, but it does not address the question of whether the “Replaced” hypothesis is better than the existing linear encoding models (the “Summed” hypothesis). We wanted to know the prevalence of “Replaced” phenomenon, not just whether it existed. That is a higher threshold of evidence to cross, and only one-third of neurons crossed it.

That said, we tested a fairly restrictive form of the “Replaced” hypothesis, where the prediction of neuronal firing at one time point was instantaneously replaced with another firing rate, usually leading to a sharp drop in predicted firing rate back to baseline. This was done deliberately, because it helped ensure that the neurons found to be better “Replaced” were not simply being over-fit. However, it did mean that other neurons might be well-described with the “Replaced” hypothesis using a more gentle transition. Thus the number of “Replaced” neurons might be underestimated.

We also found that the firing of neurons better fit by the “Replaced” hypothesis was more likely to lag velocity than lead it (see **Figure 9**). This suggests that this population is monitoring on-going movement as opposed to causally driving it. However, it is unclear why these lagging neurons would shut-off during the target jump. The simplest interpretation is that the firing of these lagging neurons is related to incoming sensory information, but that this neuronal firing is inhibited after the target jump. This might be analogous to saccadic suppression, where sensory responses are dampened in primary visual cortex during a saccade (Vallines and Greenlee, 2006) to avoid the trouble of processing a blurry image. Perturbation experiments during voluntary movement have suggested that the proprioceptive effects on MI neurons are highly attenuated during large ballistic movements as compared to finer movement adjustments (Evarts and Fromm, 1977). Therefore, modulation in this “sensory” population of MI neurons might be expected to temporarily shut-off during the early ballistic portion of the corrective movement.

Another possibility is these “Replaced” neurons are participating in a larger cortical network which monitors of the consequences of motor actions, particularly whether or not the action is successful. For example, the anterior cingulate cortex (ACC) is known to have projections to MI cortex (Dum and Strick, 1991), and the ACC has been implicated in the monitoring of the consequences of one’s actions. One proposal is that the ACC encodes the “surprise related to the non-occurrence of a predicted event” (Alexander and Brown, 2011). Neurons in the ACC have been found that show an error-related phasic increase in firing rate peaking around 200 ms after the initiation of an inappropriate saccade (Ito et al., 2003). If these neurons (or similar neurons) directly inhibited the pool of “Replaced” neurons described here, it would explain the sudden drop in firing rate following a target jump.

We focused on kinematics for this study, because our single-trial “Replaced” prediction relied on a kinematic decomposition assuming summation of the original movement and a second corrective movement. One drawback of this focus is that we did not record forces, torques, or muscle activity. The lack of force data means we were unable to investigate alternatives to kinematic summation, such as the proposal that a target jump elicits a stereotyped force pulse made in the direction of the new target (Massey et al., 1986).

Another limitation is that we excluded a fair number of neurons from analysis. We excluded 190 of 553 neurons (34%) for having a predictive encoding lag greater than +175 ms. This was done purposely to exclude neurons firing in relation to the previous target appearance (Reimer and Hatsopoulos, 2010), rather than anticipation of future velocity. We did not want to consider target encoding effects here. We also excluded an additional 81 of 553 neurons (15%) because they were not well fit by the linear encoding model. However, such neurons might still encode relevant information, particularly if they use a temporal rather than a rate code. By focusing on the two main classes of neurons, “Summed” and “Replaced,” we are underestimating the heterogeneity of neural coding.

We used the double-step reaching paradigm in these experiments to reliably induce corrections, so that we could average across trials with similar corrections. However, we view this as a model system for corrections and motor variability in general. Corrective submovements are often observed when reaches require accuracy (Crossman and Goodeve, 1983; Milner and Ijaz, 1990; Novak et al., 2000) or when infants are first learning to reach (Berthier, 1997). We predict that these “Replaced” neurons would also show a sharp change in firing rate for these naturally occurring corrections as well as those resulting from a target jump. Decomposing submovements in naturalistic movement is a difficult problem if the exact nature of the submovement is uncertain (Krebs et al., 1999). However, unpredictable gain or rotation perturbations could be applied on a single-trial basis to reliably induce corrections without the need for a sudden shift in visual target location.

The existence of a subpopulation of “Replaced” neurons is relevant for the design of brain machine interfaces. Standard techniques, such as the linear filter (Serruya et al., 2002) or the Kalman filter (Wu et al., 2002), tend to only look at the neuronal firing with leading encoding delays. This means such techniques may ignore the lagging “Replaced” neurons entirely. However, this subpopulation might be used to identify the presence of a correction, which would indicate the need for a dramatic adjustment to the estimate of hand position. Additionally, the “Replaced” subpopulation could be used to identify trials where a correction was necessary, which could be used as a teaching signal in an adaptive decoding algorithm. If the “Replacement” phenomenon is replicated in situations involving more natural corrections, then we should be able to leverage the information they contain to make brain machine interface algorithms more accurate and easier to control during real-time, closed loop control.

## Conflict of Interest Statement

The authors declare that the research was conducted in the absence of any commercial or financial relationships that could be construed as a potential conflict of interest.

## References

[B1] Alexander W. H. Brown J. W. (2011). Medial prefrontal cortex as an action-outcome predictor. *Nat. Neurosci.* 14 1338–1344 21926982 10.1038/nn.2921 PMC3183374

[B2] Archambault P. S. Caminiti R. Battaglia-Mayer A. (2009). Cortical mechanisms for online control of hand movement trajectory: the role of the posterior parietal cortex. *Cereb. Cortex* 19 2848–2864 19359349 10.1093/cercor/bhp058

[B3] Archambault P. S. Ferrari-Toniolo S. Battaglia-Mayer A. (2011). Online control of hand trajectory and evolution of motor intention in the parietofrontal system. *J. Neurosci.* 31 742–752 21228183 10.1523/JNEUROSCI.2623-10.2011 PMC6623434

[B4] Berthier N. E. (1997). “Analysis of reaching for stationary and moving objects in the human infant,” in *Neural Network Models of Cognition: Biobehavioral Foundations*, edsDonohoe J. W. Dorsel V. P. (Amsterdam:Elsevier) 283–301

[B5] Crossman E. R. Goodeve P. J. (1983). Feedback control of hand-movement and Fitts’ law. *Q. J. Exp. Psychol. A* 35 251–278 6571310 10.1080/14640748308402133

[B6] Desmurget M. Epstein C. M. Turner R. S. Prablanc C. Alexander G. E. Grafton S. T. (1999). Role of the posterior parietal cortex in updating reaching movements to a visual target. *Nat. Neurosci.* 2 563–567 10448222 10.1038/9219

[B7] Dum R. P. Strick P. L. (1991). The origin of corticospinal projections from the premotor areas in the frontal lobe. *J. Neurosci.* 11 667–689 1705965 10.1523/JNEUROSCI.11-03-00667.1991 PMC6575356

[B8] Evarts E. V. Fromm C. (1977). Sensory responses in motor cortex neurons during precise motor control. *Neurosci. Lett*. 5 267–272 19605005 10.1016/0304-3940(77)90077-5

[B9] Flash T. Henis E. (1991). Arm trajectory modifications during reaching towards visual targets. * J. Cogn. Neurosci.* 3 220–230 10.1162/jocn.1991.3.3.220 23964837

[B10] Flash T. Hogan N. (1985). The coordination of arm movements: an experimentally confirmed mathematical model. *J. Neurosci.* 5 1688–1703 4020415 10.1523/JNEUROSCI.05-07-01688.1985 PMC6565116

[B11] Georgopoulos A. P. Kalaska J. F. Caminiti R. Massey J. T. (1982). On the relations between the direction of two-dimensional arm movements and cell discharge in primate motor cortex. *J. Neurosci.* 2 1527–1537 7143039 10.1523/JNEUROSCI.02-11-01527.1982 PMC6564361

[B12] Georgopoulos A. P. Kalaska J. F. Caminiti R. Massey J. T. (1983). Interruption of motor cortical discharge subserving aimed arm movements. *Exp. Brain Res.* 49 327–340 6641831 10.1007/BF00238775

[B13] Georgopoulos A. P. Kalaska J. F. Massey J. T. (1981). Spatial trajectories and reaction times of aimed movements: effects of practice, uncertainty, and change in target location. *J. Neurophysiol.* 46 725–743 7288461 10.1152/jn.1981.46.4.725

[B14] Goodale M. A. Pelisson D. Prablanc C. (1986). Large adjustments in visually guided reaching do not depend on vision of the hand or perception of target displacement. *Nature* 320 748–750 3703000 10.1038/320748a0

[B15] Grea H. Pisella L. Rossetti Y. Desmurget M. Tilikete C. Grafton S. (2002). A lesion of the posterior parietal cortex disrupts on-line adjustments during aiming movements. *Neuropsychologia* 40 2471–2480 12417474 10.1016/s0028-3932(02)00009-x

[B16] Henis E. Flash T. (1995). Mechanisms underlying the generation of averaged modified trajectories. *Biol. Cybern.* 72 407–419

[B17] Hogan N. (1984). An organizing principle for a class of voluntary movements. *J. Neurosci.* 4 2745–2754 6502203 10.1523/JNEUROSCI.04-11-02745.1984 PMC6564718

[B18] Ito S. Stuphorn V. Brown J. W. Scall J. D. (2003). Performance monitoring by the anterior cingulate cortex during saccade countermanding. *Science* 302 120–122 14526085 10.1126/science.1087847

[B19] Krebs H. I. Aisen M. L. Volpe B. T. Hogan N. (1999). Quantization of continuous arm movements in humans with brain injury. *Proc. Natl. Acad. Sci. U.S.A.* 96 4645–4649 10200316 10.1073/pnas.96.8.4645 PMC16386

[B20] Massey J. T. Schwartz A. B. Georgopoulos A. P. (1986). On information processing and performing a movement sequence. *Exp. Brain Res.* 15 242–251

[B21] Maynard E. M. Hatsopoulos N. G. Ojakangas C. L. Acuna B. D. Sanes J. N. Normann R. A. (1999). Neuronal interactions improve cortical population coding of movement direction. *J. Neurosci.* 19 8083–8093 10479708 10.1523/JNEUROSCI.19-18-08083.1999 PMC6782478

[B22] Milner T. E. Ijaz M. M. (1990). The effect of accuracy constraints on three-dimensional movement kinematics. *Neuroscience* 35 365–374 2381512 10.1016/0306-4522(90)90090-q

[B23] Moran D. W. Schwartz A. B. (1999). Motor cortical representation of speed and direction during reaching. *J. Neurophysiol.* 82 2676–2692 10561437 10.1152/jn.1999.82.5.2676

[B24] Novak K. E. Miller L. E. Houk J. C. (2000). Kinematic properties of rapid hand movements in a knob turning task. *Exp. Brain Res.* 132 419–433 10912823 10.1007/s002210000366

[B25] Paulignan Y. MacKenzie C. Marteniuk R. Jeannerod M. (1991). Selective perturbation of visual input during prehension movements. 1. The effects of changing object position*. Exp. Brain Res.* 83 502–512 10.1007/BF00229827 2026193

[B26] Pelisson D. Prablanc C. Goodale M. A. Jeannerod M. (1986). Visual control of reaching movements without vision of the limb. II. Evidence of fast unconscious processes correcting the trajectory of the hand to the final position of a double-step stimulus*. Exp. Brain Res.* 62 303–311 10.1007/BF00238849 3709715

[B27] Prablanc C. Martin O. (1992). Automatic control during hand reaching at undetected two-dimensional target displacements. *J. Neurophysiol.* 67 455–469 1569469 10.1152/jn.1992.67.2.455

[B28] Reichenbach A. Bresciani J. P. Peer A. Bulthoff H. H. Thielscher A. (2011). Contributions of the PPC to online control of visually guided reaching movements assessed with fMRI-guided TMS. *Cereb. Cortex* 21 1602–1612 21084453 10.1093/cercor/bhq225 PMC3116739

[B29] Reimer J. Hatsopoulos N. G. (2010). Periodicity and evoked responses in motor cortex. * J. Neurosci.* 30 11506–11515 20739573 10.1523/JNEUROSCI.5947-09.2010 PMC2952881

[B30] Reina G. A. Moran D. W. Schwartz A. B. (2001). On the relationship between joint angular velocity and motor cortical discharge during reaching. *J. Neurophysiol.* 85 2576–2589 11387402 10.1152/jn.2001.85.6.2576

[B31] Rousche P. J. Normann R. A. (1992). A method for pneumatically inserting an array of penetrating electrodes into cortical tissue. * Ann. Biomed. Eng.* 20 413–422 1510293 10.1007/BF02368133

[B32] Schwartz A. B. Kettner R. E. Georgopoulos A. P. (1988). Primate motor cortex and free arm movements to visual targets in three-dimensional space. I. Relations between single cell discharge and direction of movement*. J. Neurosci.* 8 2913–2927 10.1523/JNEUROSCI.08-08-02913.1988 PMC6569414 3411361

[B33] Serruya M. D. Hatsopoulos N. G. Paninski L. Fellows M. R. Donoghue J. P. (2002). Instant neural control of a movement signal. * Nature* 416 141–142 11894084 10.1038/416141a

[B34] Soechting J. F. Lacquaniti F. (1983). Modification of trajectory of a pointing movement in response to a change in target location. * J. Neurophysiol.* 49 548–564 6834087 10.1152/jn.1983.49.2.548

[B35] Vallines I. Greenlee M. W. (2006). Saccadic suppression of retinotopically localized blood oxygen level-dependent responses in human primary visual area V1. *J. Neurosci.* 26 5965–5969 16738238 10.1523/JNEUROSCI.0817-06.2006 PMC6675218

[B36] Wang W. Chan S. S. Heldman D. A. Moran D. W. (2007). Motor cortical representation of position and velocity during reaching. *J. Neurophysiol.* 97 4258–4270 17392416 10.1152/jn.01180.2006

[B37] Wu W. Black M. J. Gao Y. Bienenstock E. Serruya M. Donoghue J. P. (2002). “Inferring hand motion from multi-cell recordings in motor cortex using a Kalman filter,” in *SAB’02-Workshop on Motor Control in Humans and Robots: On the Interplay of Real Brains and Artificial Devices*, August 10, 2002, Edinburgh, 66–73

